# Impact of consuming green and yellow vegetables on the depressive symptoms of junior and senior high school students in Japan

**DOI:** 10.1371/journal.pone.0211323

**Published:** 2019-02-07

**Authors:** Mami Tanaka, Kenji Hashimoto

**Affiliations:** Center for Forensic Mental Health, Chiba University, Chiba, Japan; Hamamatsu University School of Medicine, JAPAN

## Abstract

Adolescent depression is a significant public health concern. Although skipping breakfast is associated with depressive symptoms in adolescents, the effects of dietary patterns on their depressive symptoms remain unknown. Therefore, this study aims to determine whether dietary patterns are associated with depressive symptoms among junior and senior high school students in Japan. A total of 441 junior high school students and 417 senior high school students participated in this study. The Center for Epidemiologic Studies Depression scale (CES-D) was used to measure the participants’ depressive symptoms. We surveyed the participants’ breakfast consumption pattern, as well as their general dietary patterns (meat, fish, green and yellow vegetables, milk and dairy products, and fruits), using a self-report questionnaire. The results indicated that the senior high school students had a significantly higher CES-D score than the junior high school students. We found negative and significant partial correlations between regular consumption of breakfast and depressive symptoms, and between regular consumption of green and yellow vegetables and depressive symptoms in both junior and senior high school students, after controlling for age, sex, and sleep duration. Furthermore, a one-way analysis of covariance (ANCOVA) revealed that adolescents who consumed green and yellow vegetables every day (one or more times per day) had significantly lower depressive symptoms than those from the “Never/1–2 times a week” group. The findings of this study reveal that the regular consumption of green and yellow vegetables is associated with lower depressive symptoms in adolescents, that is, the consumption of green and yellow vegetables may be vital in the context of adolescents’ mental health.

## Introduction

At the global level, 10%–20% of children and adolescents experience mental disorders, and 50% of these mental illnesses begin by the age of 14. In addition, as much as 75% of these illnesses begin when people are in their mid-20s [1]. Psychological factors are the leading cause of mental disability in young people at the global level. If left untreated, mental disabilities may affect children’s development adversely, including their academic capabilities, as well as their capacity to live fulfilling and productive lives. This is because the human brain continues to develop throughout childhood and adolescence [2, 3]. Children with mental disorders face major challenges; they tend to be stigmatized, isolated, and discriminated against. They also typically lack access to health care and education [1]. In particular, depression is the most common mental health disorder among young people [1, 4, 5].

Regular consumption of breakfast is important for the health and development of children and adolescents. For example, the consumption of breakfast has been associated with better mental health in adults [6], young adults [7], and adolescents [8, 9, 10, 11]. Additionally, regular consumption of breakfast is linked to reduced stress and lower infection rates, both of which are concurrent with reduced cortisol activity [12]. Furthermore, junior high school students who do not consume breakfast regularly tend to be poor sleepers [13]. In contrast, adolescents who consume breakfast regularly tend to perform better academically, whereas those who skip breakfast tend to perform poorly [14, 15]. Therefore, it can be stated that the regular consumption of breakfast has a high impact on adolescents’ mental health. Nonetheless, it must be noted that other factors may also influence the association between the regular consumption of breakfast and mental distress in adolescents.

A systemic review and meta-analysis of the relationship between dietary patterns and depression indicated that a high intake of fruits, vegetables, fish, and whole grains may be associated with a reduced risk of depression [16]. A more recent meta-analysis has shown that a high intake of fruits, vegetables, whole grains, fish, olive oil, low-fat dairy products, and antioxidants coupled with a low intake of animal food, may be associated with a decreased risk of depression [17]. Conversely, a dietary pattern characterized by high consumption of red and/or processed meat, refined grains, sweets, high-fat dairy products, butter, potatoes, and high-fat gravy coupled with a low intake of fruits and vegetables, is associated with an increased risk of depression [17]. Therefore, healthy dietary patterns may reduce the risk of depression. However, there is little published information on the relationship between daily food consumption and mental health in adolescents.

Therefore, this study aims to examine the relationship between food consumption and mental distress in adolescents. First, we examined whether regular breakfast consumption is associated with depressive symptoms in junior and senior high school students in Japan. Second, we examined whether particular dietary patterns (e.g., meat, fish, green and yellow vegetables, milk and dairy products, and fruits) are associated with depressive symptoms in junior and senior high school students.

## Methods

### Participants

This study is an anonymous, cross-sectional survey involving Japanese adolescents in junior high school (grades 7–9, mean age = 13.98 years old, *SD* = .86) and senior high school (grades 10–12, mean age = 17.09 years old, *SD* = .88). The survey was conducted between September 2016 and March 2017 using a self-report questionnaire. We requested the cooperation of the principals of public junior and senior high schools in Fukuoka prefecture for this study. In addition, we requested the cooperation of the principals of public and private junior and senior high schools in Osaka prefecture. The students’ parents were informed of the research project through a letter; they were requested to notify the school if they did not want their children to participate in this study. Students were also given the choice to opt out on the day of the survey. In total, 858 junior and senior high school students (389 boys and 469 girls, mean age = 15.49 years old, *SD* = 1.78) participated in the survey. Teachers were requested to inform the students that participation in the study was entirely voluntary; they were also requested to urge the students to respond honestly—about their willingness to participate, as well as in the self-report questionnaire. Anonymous questionnaires were distributed to the students during school hours. The protocol of the study was approved by the ethics committees of the Graduate School of Medicine and School of Medicine, Chiba University (permission number: 288 on August 8, 2016).

### Measures

#### Breakfast intake

We used the following question to assess how frequently the participants ate breakfast: “How often do you eat breakfast?” The question had five response options: “never,” “1–2 times a week,” “3–4 times a week,” “5–6 times a week,” and “every day.” This presentation of breakfast intake is consistent with prior studies (e.g., [14, 18]). These options were further collapsed into three categories—“Never/1–2 times a week,” “3–6 times a week,” and “every day”—during our analysis to produce categorical data.

#### Dietary intake

To assess the typical dietary intake, we used food groups in the Japanese food guide spinning top, including meat (beef, pork, and chicken), fish, green and yellow vegetables (cabbage, broccoli, carrots, and pumpkin), milk and dairy products, and fruits, which is a tool to help people implement the “Dietary Guidelines for Japanese” [19]. In a study conducted among adults in Europe, Australia, and West Asia, an especially high consumption frequency of fish, fruits, and vegetables was correlated with lower probability of depressive symptoms (e.g., [20, 21]). In the current study, the participants’ dietary intake was assessed using questions to gauge their usual frequency of consumption, for example, they were asked the following: “How many days per week do you usually consume meat, fish, green and yellow vegetables, milk and dairy products, or fruits?” Answers were coded as “never,” “1–2 days a week,” “3–4 days a week,” “5–6 days a week,” and “one or more times per day.” These options were further collapsed into the following three categories during our analysis to produce categorical data: “Never/1–2 times a week,” “3–6 times a week,” and “one or more times per day.”

#### Depressive symptoms

The Center for Epidemiologic Studies Depression scale (CES-D) was used to measure depressive symptoms [22]. The CES-D is a short, self-report scale designed to measure depressive symptoms in the general population. The 20 items of the scale measure symptoms associated with depression; the items have also been validated against longer scales. The participants were asked to rate how frequently specific symptoms had occurred during the previous week on a four-point scale (0 = less than 1 day per week, 1 = 1–2 days a week, 2 = 3–4 days a week, and 3 = over 5–7 days a week). The total score ranged from 0 to 60, and a higher score indicates more severe depression. The Japanese version has been shown to be a reliable and valid instrument [23]. The participants with a CES-D score of ≧ 16 were regarded as depressed. The Cronbach’s alpha coefficient of the total score was .89.

#### Other variables

The participants were asked to indicate their age, sex, and sleep duration, which were treated as covariates. Barring a few exceptions, the prevalence, incidence, and morbidity risk of depressive disorders are higher in females than in males, beginning in mid-puberty and persisting through adulthood life [24, 25]. Moreover, sleep plays an important role in emotion regulation and lack of sleep can contribute to psychological problems such as depression, anxiety, and anger [26]. Clinical and epidemiological evidence has demonstrated a strong relationship between sleep and depression [27]. Weekday sleep duration information was generated by the following question: “How many hours of actual sleep do you get at night (average hours for one night)?” We categorized respondents into five groups: < four hours/night, four or more but less than five, six or more but less than seven, seven or more but less than eight, and eight or more.

### Statistical analysis

The junior and senior high school students by sex were compared for breakfast consumption, dietary intake (meat, fish, green and yellow vegetables, milk and dairy products, and fruits), and depressive symptoms variables using the one-way analysis of variance (ANOVA). When a significant difference was detected by the ANOVA, a post-hoc test of the Tukey’s honest significance test was performed. In addition, we calculated the partial correlations, controlling for age, sex, and sleep duration, between breakfast consumption, dietary patterns, and depressive symptoms among junior and senior high school students. To analyze the data thus obtained and to compare the three groups’ depressive symptoms scores (“Never/1–2 times a week,” “3–6 times a week,” and “every day [or one or more times per day]”) in relation to the frequency of breakfast consumption and dietary intake, we performed a one-way ANCOVA and a Bonferroni correction on post-hoc multiple comparisons. Statistical tests were performed using SPSS for Windows version 22. For all tests, a two-tailed *p* < .05 was considered statistically significant.

## Results

### Characteristics of the study population

Table 1 presents the characteristics of the participants—Japanese junior and senior high school students by sex. To compare the four groups (junior high school boys, junior high school girls, senior high school boys, and senior high school girls) in relation to the frequency of breakfast consumption, dietary intake, and depressive symptoms scores, we performed a one-way ANOVA and a Tukey’ s honest significance test on post-hoc multiple comparisons. One-way ANOVA analysis of breakfast consumption and consumption of milk and dairy products revealed significant differences among the four groups (*F*(3, 854) = 6.12, *p* < .001; *F*(3, 854) = 23.66, *p* < .001, respectively). The post-hoc Tukey’s test showed that junior high school boys and girls had significantly higher breakfast consumption total score compared with senior high school boys and girls (*p* < .01, 05, respectively), and that senior high school girls had a significantly lower consumption of milk and dairy products total score than the other three groups (*p* < .01, respectively). There were no differences between junior and senior high school students by sex in terms of meat, fish, green and yellow vegetables, and fruits consumption.

**Table 1:** Descriptive statistics and group differences in breakfast consumption, dietary intake, and depressive symptoms.

	Junior high school (*n* = 441)				Senior high school (*n* = 417)				ANOVA	
	Boys (*n* = 224)		Girls (*n* = 197)		Boys (*n* = 145)		Girls (*n* = 272)		*F*	post-hoc test of the Tukey’s honest significance test
	*Mean*	*SD*	*Mean*	*SD*	*Mean*	*SD*	*Mean*	*SD*		
Age	13.98	.89	13.99	.83	17.00	.95	17.13	.84	-	-
Breakfast consumption	4.73^a^	.80	4.76^a^	.76	4.41^b^	1.23	4.50^b^	1.09	6.12***	a > b
Meat consumption	3.74	1.01	3.68	1.03	3.80	.97	3.65	1.09	.76	-
Fish consumption	2.75	.96	2.63	.95	2.92	1.14	2.74	1.13	2.16	-
Green and yellow vegetables consumption	3.74	1.12	3.76	1.06	3.80	1.06	3.83	1.07	.33	-
Milk and dairy products consumption	3.88^a^	1.11	3.60^b^	1.20	3.40^c^	1.22	3.01^d^	1.28	23.66***	a > c > d, b > d
Fruits consumption	2.96	1.30	3.11	1.24	2.81	1.34	2.81	1.37	2.43	-
Depressive symptoms	12.15^a^	7.88	12.57^a^	7.12	21.28^b^	10.63	21.88^b^	10.83	72.92***	a < b

*Note*. ANOVA = one-way analysis of variance.

***p < .001.

**Fig 1:**
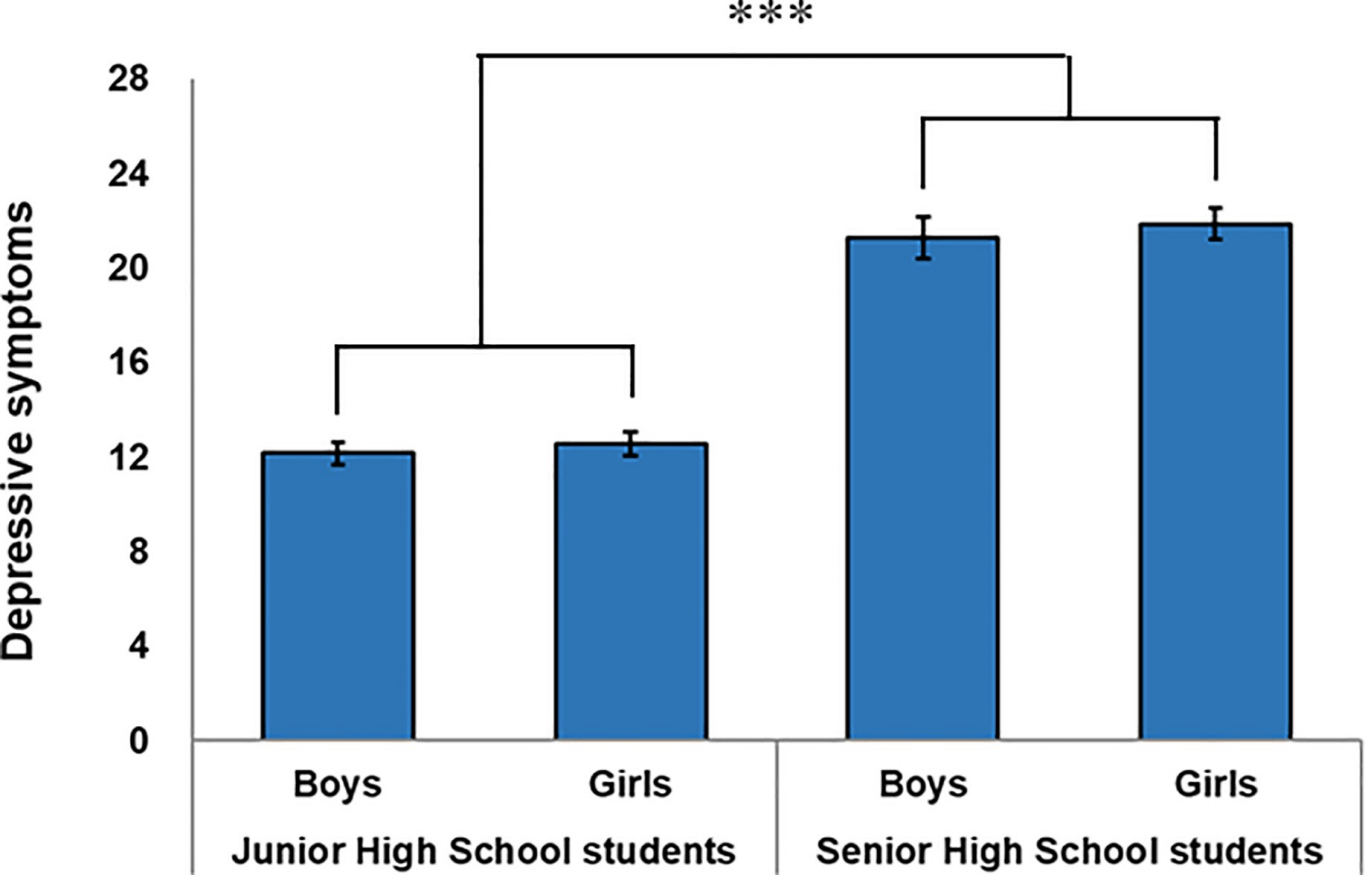
Difference in depressive symptoms between junior and senior high school students by sex. Rating scales regarding depression symptoms where one-way ANOVA and post-hoc Tukey’s honest significance test showed significant differences among four groups. Error bars represent standard error of the mean. ***p < .001.

Fig 1 indicates the rating scale for depressive symptoms where the one-way ANOVA and subsequent analysis showed significant differences among the four groups (*F*(3, 854) = 72.92, *p* < .001). The post-hoc Tukey’s test showed that the senior high school students had a significantly higher CES-D score than the junior high school students (*p* < .001, respectively) (Fig 1). In addition, we also found a positive and significant correlation between age and CES-D scores among the junior and senior high school students (*r* = .39, *p* < .001). The data suggest that adolescents tend to develop depressive symptoms at different ages. Sex-based differences in depressive symptoms revealed that girls were more depressed than boys (*r* = .12, *p* < .001). We also found a negative and significant correlation between sleep duration and CES-D scores (*r* = −.28, *p* < .001). Therefore, the participants’ ages, sex, and sleep durations were treated as covariates.

### Partial correlation analysis

We calculated the partial correlations, controlling for age, sex, and sleep duration, between depressive symptoms, breakfast consumption, and dietary patterns (Table 2). The consumption of breakfast was found to be negatively and significantly correlated with depressive symptoms in both junior and senior high school students (*r* = −.17, *p* < .01, *r* = −.15, *p* < .01, respectively). Furthermore, the consumption of green and yellow vegetables was also found to be negatively and significantly correlated with depressive symptoms in both junior and senior high school students (*r* = −.15, *p* < .01, *r* = -.11, *p* < .05, respectively). However, the consumption of fish was found to be negatively and significantly correlated with depressive symptoms only in the junior high school students (*r* = −.11, *p* < .05).

**Table 2:** Partial correlations between depressive symptoms and consumption of breakfast and dietary intake.

	Depressive symptoms	
	Junior high school students	Senior high school students
Breakfast consumption	-.17**	-.15**
	[-.26, -.08]	[-.25, -.06]
Meat consumption	-.03	-.04
	[-.12, .06]	[-.13, .06]
Fish consumption	-.11*	.07
	[-.20, -.01]	[-.03, .17]
Green and yellow vegetables consumption	-.15**	-.11*
	[-.24, -.06]	[-.21, -.02]
Milk and dairy products consumption	-.02	-.03
	[-.11, .07]	[-.12, .07]
Fruits consumption	-.08	-.02
	[-.17, .01]	[-.12, .07]

*Note*. Partial Correlation coefficients were controlled for age, sex, and sleep duration. 95% confidence intervals are reported in brackets.

*p < .05 and

**p < .01.

### Consumption of breakfast, consumption of green and yellow vegetables, and depressive symptoms

**Fig 2:**
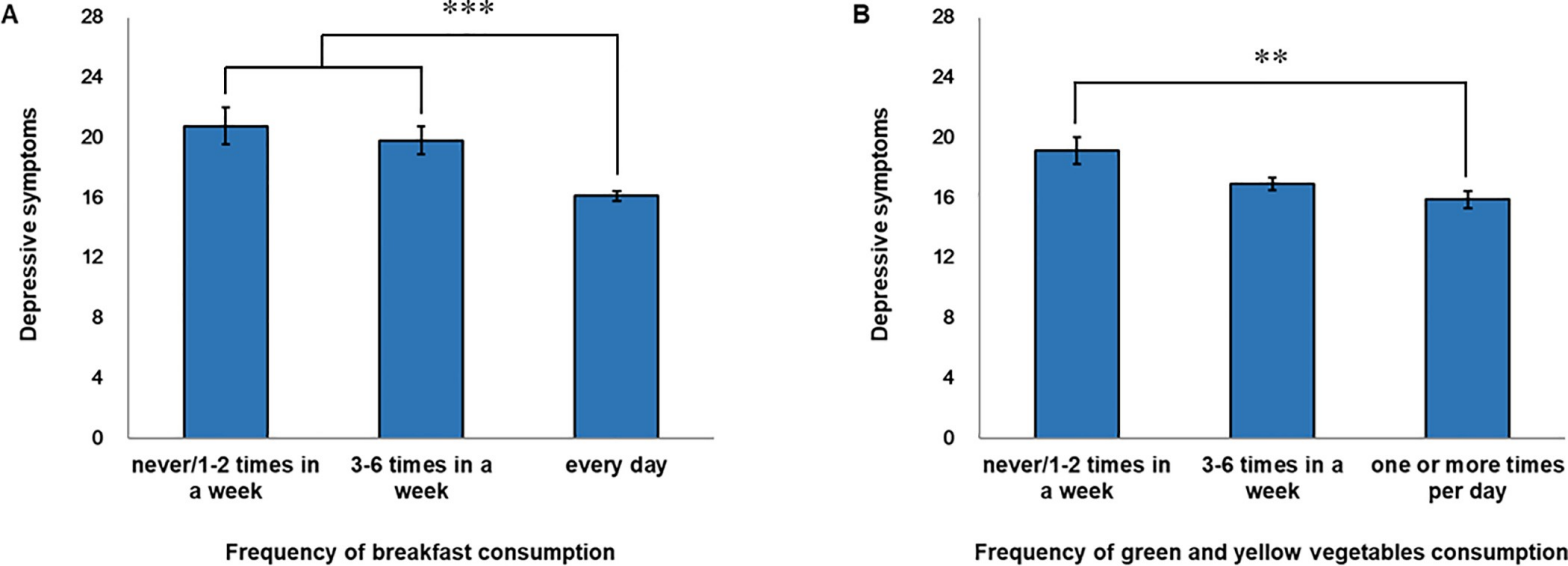
Difference in depressive symptoms by frequency of breakfast consumption and green and yellow vegetables consumption. (A) Rating scales regarding depression symptoms where ANCOVA and a Bonferroni correction to post-hoc multiple comparisons showed significant differences among three groups of breakfast consumption. (B) Rating scales showing depression symptoms where ANCOVA and a Bonferroni correction to post-hoc multiple comparisons showed significant differences among three groups of green and yellow vegetables consumption. After adjusting for covariates, age and sleep duration. Error bars represent standard error of the mean. **p < .01 and ***p < .001.

To compare depressive symptoms when controlling for age in the groups “Never/1–2 times a week,” “3–6 times a week,” and “every day (or one or more times per day)” in terms of the junior and senior high school students’ frequency of breakfast consumption and green and yellow vegetables consumption, we performed a one-way ANCOVA with age and sleep duration as covariates and depressive symptoms as the dependent variable (because the slope of the regression line of sex was not confirmed [*B* = .59, *t* = .91, *p =* .37 for breakfast eating; *B* = .51, *t* = .78, *p =* .44 for green and yellow vegetables], we performed a one-way ANCOVA with age and sleep duration as a covariate excepting sex). First, we observed that the regression line between age and depressive symptoms and that between sleep duration and depressive symptoms in each group were parallel (for breakfast eating, *F* (2, 849) = .18, *p =* .83, *F* (2, 849) = 44, *p =* .64, respectively; for green and yellow vegetables, *F* (2, 849) = 1.96, *p =* .14, *F* (2, 849) = .24, *p =* .79, respectively). Because the slope of the regression line of age and sleep duration was also confirmed (for breakfast eating, *B* = 1.83, *t* = 9.52, *p* < .001, *B* = −1.76, *t* = −4.81, *p* < .001, respectively; for green and yellow vegetables, *B* = 1.95, *t* = 10.09, *p* < .001, *B* = −1.78, *t* = −4.84, *p* < .001, respectively), covariance analysis was the most effective choice. The ANCOVA indicated that breakfast consumption had a significant main effect (*F* (2, 853) = 12.13, *p* < .001); consumption of green and yellow vegetables was also found to have a significant main effect (*F* (2, 853) = 4.82, *p* < .01) among the three subgroups. A Bonferroni correction to post-hoc multiple comparisons showed that the depressive symptoms of adolescents from the eat breakfast “Never/1–2 times a week” and “3–6 times a week” groups was significantly higher than that of the “every day” group (*p* < .001) (The panel A in Fig 2). A Bonferroni correction to post-hoc multiple comparisons showed that the depressive symptoms of adolescents from the group that ate green and yellow vegetables “Never/1–2 times a week” was significantly higher than that of the “one or more times per day” group (*p* < .01) (The panel B in Fig 2).

## Discussion

In this study, we examined the effects of eating breakfast and dietary patterns on junior high school students (*n* = 441) and senior high school students (*n* = 417) in two cities in Japan. First, the senior high school students had significantly higher CES-D scores than the junior high school students, suggesting that depressive symptoms may be associated with adolescents’ age. Second, the consumption of breakfast was negatively correlated with depressive symptoms in both junior and senior high school students. Third, the consumption of green and yellow vegetables was also negatively correlated with depressive symptoms in both junior and senior high school students. These findings suggest that the consumption of breakfast and green and yellow vegetables has an impact on the depressive symptoms of Japanese adolescents.

The study revealed that the senior high school students had higher depressive symptoms than the junior high school students. In general, the prevalence estimates of depression indicate that adolescents (approximately 13–18 years) and young adults (approximately 19–26 years) tend to have higher depressive symptoms than school children (approximately 7–12 years) [28]. The National Institute of Mental Health reported that 12.8% of adolescents aged between 12 and 17 years in the United States had at least one major depressive episode, and that the prevalence of major depressive episodes increased with age within their age range [29]. The present study also showed that the depressive symptoms score increased with the adolescents’ age. In particular, the mean scores of depressive symptoms of those over 16 years of age were higher than the cut-off value of the CES-D score (CES-D score of ≧ 16). Although the reasons for this difference are unknown, one reason may be that the transition from childhood to adolescence to early adulthood coincides with major biological, social, and psychological changes. It appears that senior high school students (over 16 years) experience more stressful events than junior high school students. For example, late adolescents tend to face intense moodiness and parent-child conflicts, peer conflicts, and conflicts with friends; they also tend to spend more time with friends. Additionally, they typically experience distress related to romantic relationships and difficulties in objective and subjective school performance [30, 31, 32]. Future studies should attempt to investigate the role of psychosocial factors, such as life events related to social relationships and school performance and personality, in relation to daily food consumption, mental distress, and health during the transition from early adolescence to late adolescence.

This study demonstrated that the consumption of breakfast was associated with lower depressive symptoms in adolescents, a finding that is consistent with previous reports [8, 9, 10, 11]. Interestingly, Itani, Kaneita, Munezawa, Ikeda, Osaki, Higuchi, Kanda, Nakagome, Suzuki, and Ohida [33] reported a significant association between skipping breakfast and anger/impulsivity in both junior high school students (*n* = 10,955) and senior high school students (*n* = 5,115) in Japan. Considering the key role breakfast plays in mental health in children and adolescents, it seems that regular breakfast consumption has a high impact on adolescents’ mental health. However, this study does not examine the detailed dietary patterns of the participants’ breakfast. Therefore, it is also important to conduct a more detailed study of the impact of the dietary pattern of breakfast on adolescents’ mental health.

Interestingly, we found that the consumption of green and yellow vegetables was negatively and significantly associated with depressive symptoms in the junior and senior high school students. In addition, the analysis comparing eating frequency showed that adolescents who consumed green and yellow vegetables every day (one or more times per day) had significantly lower depressive symptoms than those from the “Never/1–2 times a week” group, with the former’s mean score also being lower than the cut-off value of the CES-D score. The side dish-intake pattern of Japanese people is typically characterized by the high intake of seaweeds, mushrooms, green and yellow vegetables, seafood, light-colored vegetables, potatoes, and pickles. This intake pattern was found to be associated with lower rates of depressive states in Japanese university students (*n* = 240) [34]. Therefore, it is likely that the consumption of green and yellow vegetables may lower the depressive symptoms of Japanese adolescents. Toyomaki, Koga, Okada, Nakai, Miyazaki, Tamakoshi, Kiso, and Kusumi [35] focused on the association between dietary patterns and various mental health measures among healthy middle-aged Japanese people using cluster analysis. Those who predominantly consumed vegetables and fruits showed increases in several aspects of subjective well-being, whereas those who consumed grains less frequently showed higher impulsive behavior [35]. This study suggests that traditional Japanese dietary patterns can have a positive impact on mental health. Although this study does not focus directly on the relation between fruits and depressive symptoms, a negative and significant correlation was observed between the consumption of fish and the depressive symptoms of junior high school students alone. Based on these results, as well as the findings reported by previous studies focusing on adults [17, 36], the consumption of fish may be important for the prevention of adolescent depression, especially early adolescent depression.

Women whose diet includes more foods that trigger inflammation—such as sugar-sweetened or diet soft drinks, refined grains, red meat, and margarine—and fewer foods that limit inflammation—such as wine, coffee, olive oil, and green leafy and yellow vegetables—have a 41% greater risk of being diagnosed with depression than those who predominantly consume the latter [37]. This study suggests that an inflammatory dietary pattern is associated with a higher depression risk. In the preclinical study, we reported that pretreatment with sulforaphane (a potent anti-inflammatory natural compound found in cruciferous vegetables) significantly blocked the increase in the serum tumor necrosis factor-α (TNF-α) level after a single administration of lipopolysaccharide (LPS). Furthermore, pretreatment with sulforaphane also blocked depression-like phenotypes in mice after LPS administration [38]. Moreover, the intake of glucoraphanin (a glucosinolate precursor of sulforaphane) during late childhood and adolescence could prevent the onset of LPS-induced depression-like behaviors and dendritic spine changes in the brain regions during adulthood [38]. In addition, we reported that the intake of glucoraphanin during late childhood and adolescence might prevent the onset of depression-like phenotype in mice after chronic social defeat stress [39]. These preclinical findings suggest that the dietary intake of sulforaphane-rich vegetables has prophylactic effects on inflammation-related depressive symptoms in humans.

Several limitations of the present study need to be addressed. First, because the participants were not randomly selected, there could have been a referral bias. Therefore, a well-structured randomized study will be needed in the future. Second, although we investigated age, sex, and sleep duration, there may be other significant background variables for the relationship between dietary patterns and depressive symptoms, for example, body mass index, exercise habits, and parental income and education. Finally, because this was a cross-sectional survey, causal relationships could not be determined. A future longitudinal study will be required to examine causal relationships. In particular, it will be important to examine how dietary patterns affect adolescent depressive symptoms during the adolescent physical and mental development and growth period.

Despite these limitations, this study suggests that breakfast consumption and dietary patterns have a large impact on adolescents’ depressive symptoms. Specifically, the consumption of green and yellow vegetables may play a role in lowering the depressive symptoms of adolescents.
